# Loss of pericyte smoothened activity in mice with genetic deficiency of leptin

**DOI:** 10.1186/s12860-017-0135-y

**Published:** 2017-04-20

**Authors:** Guanhua Xie, Marzena Swiderska-Syn, Mark L. Jewell, Mariana Verdelho Machado, Gregory A. Michelotti, Richard T. Premont, Anna Mae Diehl

**Affiliations:** 1 Department of Medicine, Division of Gastroenterology, Duke University, 905 S. LaSalle Street, Snyderman Building, Suite 1073, Durham, NC 27710 USA; 2 Current address: Medical University of South Carolina, Charleston, SC 29425 USA; 3 Current address: Santa Maria Hospital, University of Lisbon, Lisbon, Portugal; 4 Current address: Metabolon Inc, Research Triangle Park, NC 27709 USA

**Keywords:** Obesity, Leptin, Hedgehog, Pericytes, Energy balance, Tissue repair

## Abstract

**Background:**

Obesity is associated with multiple diseases, but it is unclear how obesity promotes progressive tissue damage. Recovery from injury requires repair, an energy-expensive process that is coupled to energy availability at the cellular level. The satiety factor, leptin, is a key component of the sensor that matches cellular energy utilization to available energy supplies. Leptin deficiency signals energy depletion, whereas activating the Hedgehog pathway drives energy-consuming activities. Tissue repair is impaired in mice that are obese due to genetic leptin deficiency. Tissue repair is also blocked and obesity enhanced by inhibiting Hedgehog activity. We evaluated the hypothesis that loss of leptin silences Hedgehog signaling in pericytes, multipotent leptin-target cells that regulate a variety of responses that are often defective in obesity, including tissue repair and adipocyte differentiation.

**Results:**

We found that pericytes from liver and white adipose tissue require leptin to maintain expression of the Hedgehog co-receptor, Smoothened, which controls the activities of Hedgehog-regulated Gli transcription factors that orchestrate gene expression programs that dictate pericyte fate. Smoothened suppression prevents liver pericytes from being reprogrammed into myofibroblasts, but stimulates adipose-derived pericytes to become white adipocytes. Progressive Hedgehog pathway decay promotes senescence in leptin-deficient liver pericytes, which, in turn, generate paracrine signals that cause neighboring hepatocytes to become fatty and less proliferative, enhancing vulnerability to liver damage.

**Conclusions:**

Leptin-responsive pericytes evaluate energy availability to inform tissue construction by modulating Hedgehog pathway activity and thus, are at the root of progressive obesity-related tissue pathology. Leptin deficiency inhibits Hedgehog signaling in pericytes to trigger a pericytopathy that promotes both adiposity and obesity-related tissue damage.

**Electronic supplementary material:**

The online version of this article (doi:10.1186/s12860-017-0135-y) contains supplementary material, which is available to authorized users.

## Background

Obesity is a leading preventable cause of death worldwide, with increasing rates in adults and children. Multiple obesity-associated diseases (osteoarthritis, sleep apnea, diabetes, cancer, cardiovascular disease, and nonalcoholic fatty liver disease) together reduce life expectancy by almost 7 years [1]. In 2013, the American Medical Association classified obesity itself as a disease, prompting renewed efforts to clarify how obesity promotes tissue damage.

Ob, the first obesity gene to be identified [2], encodes the fat cell-derived adipokine hormone leptin. While genetic leptin deficiency is rarely a cause of human obesity, leptin resistance is common in obese patients [3]. Hence, the consequences of reduced leptin signaling are broadly relevant. Leptin-deficient ob/ob mice have been studied intensively as a model of obesity and its co-morbidities: ob/ob mice are not only hyperphagic and morbidly obese, but also dysmorphic and prone to various types of tissue damage [4, 5]. It remains unclear how deficiency of this single anorexogenic hormone profoundly disrupts functions in multiple tissues to generate this complex phenotype. One general concept is that leptin is a key component of the sensor that matches cellular energy utilization to available energy supplies. Energy-intensive activities that are feasible when energy stores are replete (e.g., tissue repair) become impossible when energy stores are depleted, so organisms restrict energy-intensive responses as energy stores dwindle. High leptin levels indicate energy abundance, while low leptin means low energy availability.

Loss of leptin should be sufficient to trigger the “energy-restricted” response in leptin-target cells, even when systemic energy depots are replete. We examined leptin signaling in two key leptin target tissues that are extremely dysfunctional in ob/ob mice: liver and white adipose tissue (WAT). Livers of leptin-deficient mice demonstrate progressive obesity-related organ dysfunction that enhances vulnerability to organ damage by becoming fatty and insulin-resistant at a young age, failing to regenerate effectively after partial hepatectomy in even early adolescence, and exhibiting defective wound healing when chronically injured by hepatotoxins in adulthood [6–8]. WAT in ob/ob mice is also very abnormal, becoming progressively populated by hypertrophic insulin-insensitive adipocytes with age, as is typical of WAT in obese humans [9]. In these leptin-target tissues, we focused on resident perivascular cells (pericytes) that wrap around the tissue microvasculature (i.e., sinusoids/capillaries, small post-capillary venules), because they are vital for tissue health and regulate a variety of responses that are often defective in obesity, such as blood flow within tissues, vascular permeability, and local wound healing responses, including inflammation, angiogenesis, matrix remodeling and tissue regeneration [10]. Importantly, pericytes in liver and WAT both produce and respond to leptin [11, 12], and thus provide systems to dissect cell-autonomous actions that are triggered by loss of leptin signaling. It is particularly noteworthy that WAT and liver pericytes orchestrate long-lived morphogenic responses that permit the entire organism to adapt to persistent changes in energy availability: WAT pericyte populations harbor adipocyte progenitors that differentiate so that adipose depots are re-configured in accordance with long-term changes in energy supply/demand [10], while liver-resident pericytes coordinate liver growth [13, 14]. Viewed from this perspective, leptin-responsive pericytes evaluate energy availability to inform tissue construction and thus are at the root of progressive obesity-related organ pathology, so we reasoned that leptin-responsive pericytes from leptin-deficient ob/ob mice should exhibit phenotypic abnormalities consistent with obesity-enhanced vulnerability to tissue damage.

Leptin exerts its actions on pericytes by directly binding to leptin receptors (ObR) [15]. Of the six receptor splice variants, only ObRb signals intracellularly by interacting with Jak2 to enhance phosphorylation/activation of Stat3 [3]. Stat3 activation appears to be essential for leptin’s overall effects on energy balance because Stat3-deficient mice and db/db mice (with a mutation in ObRb that prevents leptin activation of Jak-Stat) both are obese [16, 17]. Recently, white adipocyte precursors were shown to up-regulate the Hedgehog (Hh) pathway when JAK 1/2-Stat3 signaling was acutely inhibited, and Hh pathway activation increased UCP1 mitochondrial uncoupling to dissipate energy to compensate for the loss of Stat3 [18]. We found these results to be particularly intriguing because the Hh pathway is a developmental morphogenic signaling pathway that becomes reactivated in adulthood when tissues regenerate. Moreover, recent lineage tracing studies have revealed that resident pericyte populations in multiple tissues are marked by Hh pathway activity, and Hh signaling controls pericyte fate decisions [19]. For example, Hh pathway activation stimulates liver-resident pericytes (a.k.a. hepatic stellate cells) to acquire proliferative, migratory, and myofibroblastic traits that are necessary for liver growth and repair [20, 21]. Hh also regulates the differentiation of pericyte-derived adipocyte progenitors: high pathway activity blocks WAT development [22]. Most importantly, the Hh pathway is a highly conserved regulator of systemic energy homeostasis, and obesity results in flies, mice, and humans when Hedgehog activity is inhibited [22, 23].

Here we evaluate the hypothesis that leptin deficiency inhibits Hedgehog signaling in pericytes to trigger a pericytopathy that promotes both adiposity and obesity-related organ damage. Our findings support this concept and suggest a novel model whereby pericytes require leptin to maintain expression of the Hedgehog co-receptor, Smoothened (Smo), which controls the activities of Hedgehog-regulated Gli-family transcription factors that orchestrate gene expression programs that dictate pericyte fate. When leptin is deficient, Smo activity falls and Hh signaling is disrupted, leading to changes in gene expression that induce pericyte senescence that, in turn, compromises tissue health and promotes obesity-related organ damage.

## Results

### Leptin is required for hedgehog pathway activation in liver resident pericytes

**Fig. 1:**
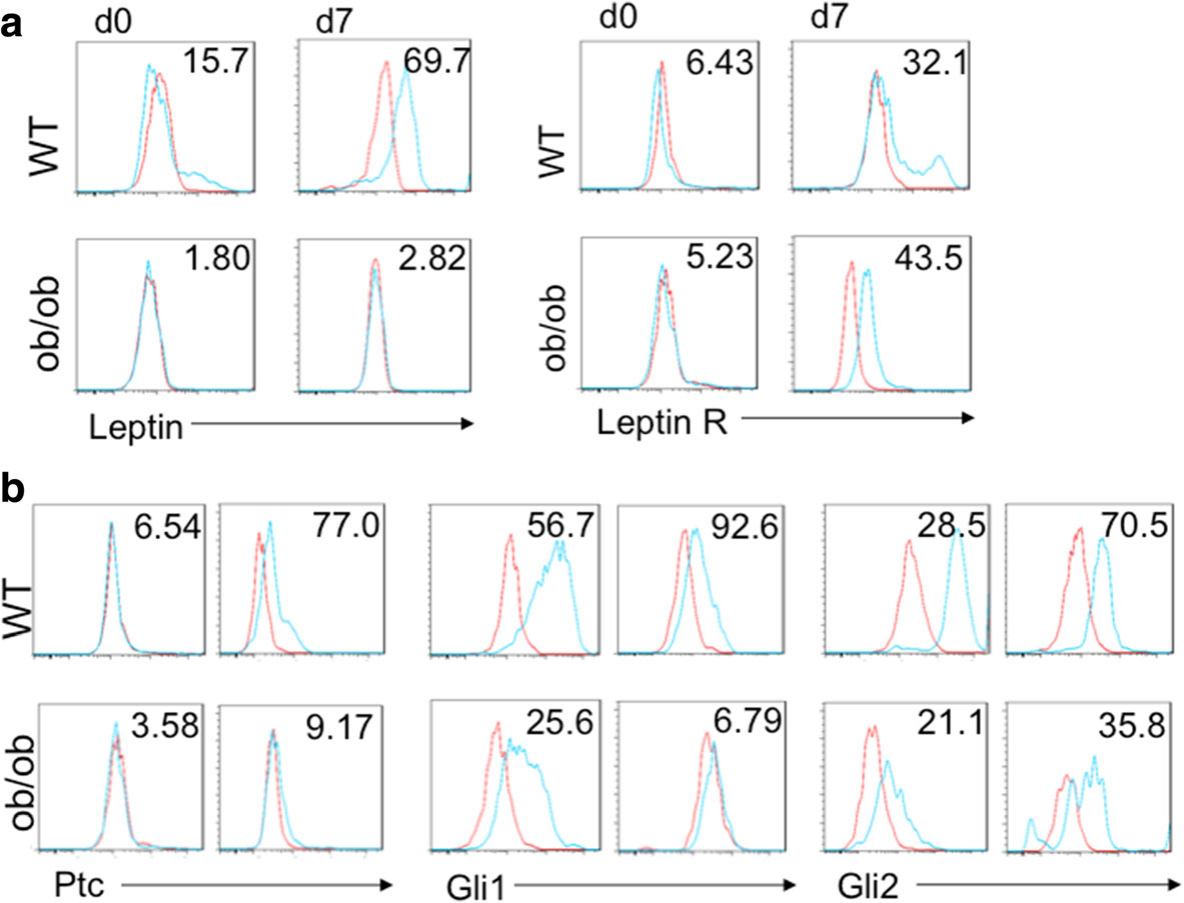
Genetic Leptin Deficiency Does Not Alter Leptin Receptor Expression But Suppresses Hh Activity in HSCs. **a**. Primary HSCs were isolated from WT mice or ob/ob mice, and cultured on plastic dishes in serum-containing medium for 2 h (d0) or 7 days (d7). FACS analysis of (*left*) Leptin and (*right*) Leptin receptor (OBRa plus OBRb) immunoreactivity in quiescent (day 0, d0) and myofibroblastic (culture day 7, d7) HSCs. *Red lines* indicate Isotype controls. **b**. FACS analysis analysis of Hh target gene products (Ptc, Gli1, Gli2) in quiescent and myofibroblastic HSC from WT and ob/ob mice. *Red lines* indicate Isotype controls

Liver-resident pericytes (also known as hepatic stellate cells, HSC) in healthy adult livers express both leptin and its receptors, and leptin signaling activity increases as quiescent (Q)- HSCs trans-differentiate to become myofibroblastic (MF)-HSCs [15, 24]. Consistent with previous reports [12, 15, 25], upregulation of both leptin and leptin receptor (ObR) expression was observed when primary mouse WT Q-HSCs transdifferentiated into MF-HSCs (Fig. 1a). In contrast, almost no leptin was detected in primary HSCs isolated from ob/ob mice, either when the cells were quiescent or myofibroblastic. Expression of leptin receptors was similar in WT and ob/ob HSCs, however (Fig. 1a).

Similar to the Leptin pathway, Hh signaling is induced during HSC trans-differentiation [20, 26, 27]. Canonical Hh signaling culminates in the activation of Glioma (Gli) transcription factors (Gli1, Gli2, Gli3) that control the expression of Hh-regulated genes. Sonic Hedgehog ligand (Shh) binds to the cell surface membrane spanning receptor, Patched (Ptc), to abrogate Ptc-mediated inhibition of the co-receptor Smoothened (Smo). Smo controls the processing and stability of the Gli factors. When Smo is inactive, Gli factors are degraded and/or processed to transcriptional repressors. Smo activation stabilizes Gli factors and promotes their nuclear accumulation to transcribe multiple Gli-regulated genes, including Gli1 itself, a key transcriptional activator of multiple other Hh-target genes, including Ptc [28]. Previously we reported that Hh pathway activity is required for leptin-mediated induction of HSC trans-differentiation [15, 20, 29]. However, it is not known if leptin is necessary for activation of the Hh pathway during HSC trans-differentiation. We compared Hh pathway activity in WT versus ob/ob HSCs. As expected, trans-differentiation of WT HSCs was accompanied by increased protein expression of Gli2 and Hh target genes (Ptc, Gli1) (Fig. 1b). In contrast, Gli2 and Hh-target genes were significantly suppressed in ob/ob HSCs, regardless of whether the cells were examined when freshly isolated or culture-activated for 7 days (Fig. 1b). Intriguingly, half of the WT HSCs expressed Gli1 protein when freshly isolated and over 90% were Gli1-positive after culture-induced activation, corroborating the notion that WT HSC populations are normally enriched with Hh-responsive pericytes that can be reprogrammed into myofibroblasts [19]. Commercially-available antibodies do not reliably demonstrate Shh in mouse cells, so we used qRT PCR to compare expression of Shh mRNA in WT and ob/ob HSC (Additional file 1: Figure S1). Compared to WT HSC, ob/ob HSC expressed significantly lower levels of Shh mRNA, both when freshly isolated and after 7 days in culture. Taken together, these data indicate that HSC require leptin to activate the Hh pathway and suggest that leptin normally activates Hh signaling in stellate cells in an autocrine fashion.

### Leptin-deficient HSC with loss of Hh activity Are prone to senescence

**Fig. 2:**
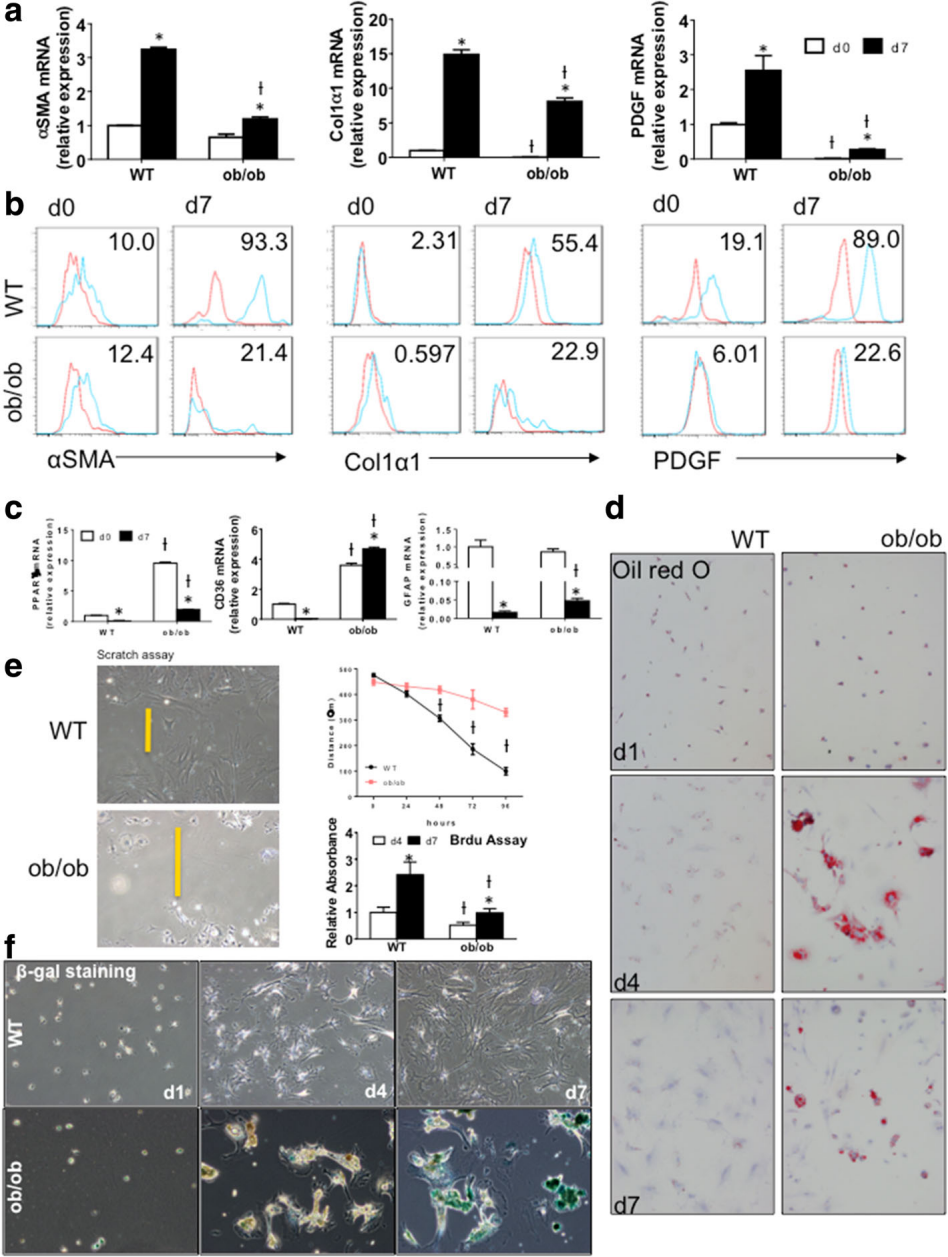
Leptin Deficiency Changes HSC Phenotype. Primary HSCs isolated from WT and ob/ob mice were cultured on plastic dishes in serum-containing medium for 2 h (d0) or 7 days (d7). **a** qRT-PCR and (**b**) FACS analysis of MF genes (αSMA, Col1α1, and PDGF), and (**c**) qRT-PCR analysis of quiescence genes (PPARγ, CD36, and GFAP) in quiescent and myofibroblastic HSCs. Results were normalized to WT d0 cells. Ɨ p < 0.05 vs WT, * p < 0.05 vs d0. n = 3. *Red lines* indicate Isotype controls. **d** Lipid content of cultured HSC at indicated days was examined by Oil Red O staining. (original magnification, ×10) (**e**) Migration and proliferation of HSCs were measured by scratch assay and BrdU assay. Results were normalized to WT d4 cells. Ɨ p < 0.05 vs WT, * p < 0.05 vs d0. n = 3. **f** Senescence was examined by β-galactosidase staining (*blue*) of HSCs cultured for 1, 4 and 7 days. (original magnification, ×10)

Having shown that Hh activity is suppressed in leptin-deficient HSCs, we next examined whether HSCs from ob/ob mice exhibit altered trans-differentiation. In WT HSCs, two well-accepted myofibroblastic markers, α-smooth muscle actin (αSMA) and collagen 1α1 (col1α1), and the fibrogenic growth factor platelet-derived growth factor (PDGF) were significantly upregulated at both mRNA and protein levels when Q-HSCs transdifferentiated into MF-HSCs (Fig. 2a, b). Although ob/ob HSCs also increased these mesenchymal markers when stimulated to become MF, expression levels were much lower in ob/ob HSCs than WT HSCs, both during quiescence and after culture-activation. In contrast, ob/ob HSCs had significantly higher mRNA expression of two quiescent stellate cell markers, peroxisome proliferator activated receptor gamma (PPARγ) and CD36 [30, 31] at both quiescent and myofibroblastic stages (Fig. 2c). Another well-acknowledged quiescent stellate cell marker, glial fibrillary acidic protein (GFAP) [32], was more highly expressed in culture-activated ob/ob HSCs than culture-activated WT HSCs (Fig. 2c). Moreover, Oil Red O staining (Fig. 2d) showed that there was more lipid accumulation in culture-activated ob/ob HSCs than in WT HSCs, consistent with the gene expression profiles of PPARγ and CD36, two factors that have important roles in lipid regulation [33]. Scratch wound and BrdU assays (Fig. 2e, f) demonstrated that migration and proliferation of ob/ob HSCs were dramatically inhibited, adding to evidence that the leptin-deficient HSC maintain a less myofibroblastic phenotype than WT HSC.

**Fig. 3:**
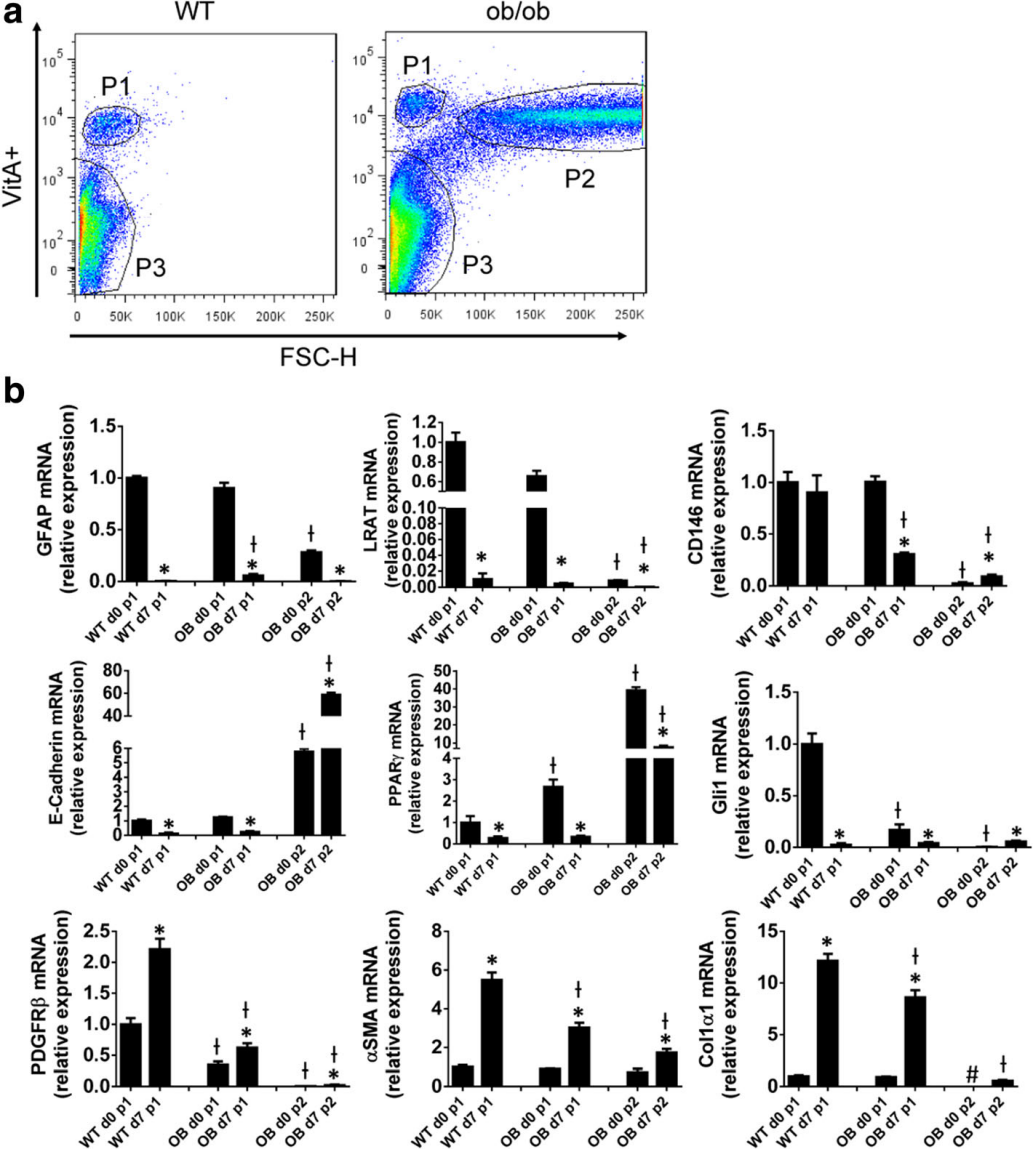
Leptin Deficiency Alters HSCs That Are Purified by Retinoid-based FACS Isolation. **a** Ultrapure HSCs were obtained by retinoid-based FACS of Nycodenz-gradient purified HSCs from WT and ob/ob mice. P1 population contains ultrapure HSCs, P2 population contains high side scatter (i.e., large), retinoid-high cells and only exists in ob/ob HSC prep, and P3 population contains cell debris and dead cells (which are removed via a 2 h cell adherence step in our standard HSC preps) (**b**) RNA was isolated from both freshly FACS sorted P1 or P2 cells (d0) or 7 day cultured FACS-sorted cells (d7), and gene expression profiles were examined by qRT-PCR. Ɨ *p* < 0.05 vs WT, * *p* < 0.05 vs d0. # non-detectable

To evaluate the possibility that these observed differences between ob/ob and WT HSCs might be due to contamination of the ob/ob HSC isolates with other cell types, ultrapure primary HSCs were obtained from additional ob/ob and WT mice according to a stringent protocol that involves an additional retinoid-based fluorescent cell sorting step that identifies Q-HSC as a discrete subpopulation of small (low side-scatter) cells with high vitamin A fluorescence [34], hereafter dubbed ultrapure HSC (P1) (Fig. 3a). Fluorescence cell sorting identifies a similar P1 pool in HSC preps isolated from ob/ob mice, as well as a larger (higher side scatter) high retinoid pool (hereafter dubbed P2) (Fig. 3a). When freshly isolated, these ultrapure WT and ob/ob P1-HSCs expressed similar mRNA levels of markers of HSC quiescence (LRAT, GFAP), pericytes (CD146), and epithelial cells (E-cadherin). However, expression of the adipogenic transcription factor, PPARγ, was nearly 3 fold greater in the ob/ob P1 HSCs than WT HSCs. PPARγ expression is negatively regulated by Gli1 [28] and consistent with this, ultrapure ob/ob HSCs expressed ~80% less Gli1 than similarly isolated WT HSC. In contrast, PDGFRβ (a Gli1-inducible gene that encodes a PDGF receptor that transduces critical growth and viability signals for HSCs and other pericytes [35, 36]) was ~80% reduced in freshly isolated P1-HSC from ob/ob mice relative to P1-HSC from WT mice (Fig. 3b). During culture-activation, ultrapure WT HSC maintained stable expression of CD146, upregulated PDGFRβ, and became myofibroblasts (as evidenced by dramatic induction of αSMA and col1α1) (*p* < 0.05 for culture-activated versus freshly-isolated HSC). In contrast, CD146 mRNA levels dropped by more than 50% in ultrapure ob/ob HSC during culture; their PDGRFβ expression remained low, and induction of aSMA and collagen was significantly suppressed (*P* < 0.05 for all markers in culture-activated ob/ob versus culture-activated WT HSC) (Fig. 3b).

The reduced expression of CD146 and PDGFRβ in cultured ob/ob HSC suggested that the leptin-deficient pericytes might be prone to exhaustion. Immunocytochemical analysis of β-galactosidase activity confirmed this hypothesis, demonstrating that culture under pro-fibrogenic conditions significantly enriched ob/ob HSC populations with senescent cells, whereas relatively little senescence occurred when WT HSC were cultured under identical conditions (Fig. 2f). Senescent-appearing cells were particularly enriched in the P2 subpopulation of the ob/ob HSC isolate that was comprised of high side-scatter (i.e., large), retinoid-high cells. This P2 high retinoid/large cell fraction expressed the highest levels of PPARγ mRNA and the lowest levels of Gli1 mRNA. Further, although similar levels of αSMA were demonstrated in this freshly isolated (day 0) retinoid-high ob/ob large cell P2 fraction and in the comparable ultrapure P1 fractions of freshly-isolated retinoid-high small WT and ob/ob HSC, col1α1 mRNA was undetectable in the large, retinoid-high ob/ob P2 cells on day 0, but easily demonstrated (and expressed at comparable levels) in the other freshly isolated (day 0) P1 HSC subpopulations (Fig. 3b). Although both WT and ob/ob HSCs were able to upregulate col1α1 during culture, this response was significantly attenuated in cultured P1 ob/ob HSC and nearly abolished in P2 ob/ob cells. Loss of col1α1 expression is a trait typical of senescent HSC [37, 38], as is increased expression of the cell cycle inhibitor p16 [39]. Levels of p16 mRNA were more than 20 fold higher in P2 ob/ob HSC on day 0 than in the freshly isolated P1 fractions of either ob/ob or WT HSC (*p* < 0.05 versus other P1 fractions). Collectively, therefore, decreased Hh activity due to leptin deficiency causes stellate cells to become prematurely senescent, limiting their ability to be re-programmed into fibrogenic myofibroblasts both in vitro and in vivo.

### Leptin-deficient HSCs release signals that alter the phenotype of hepatocytes

**Fig. 4:**
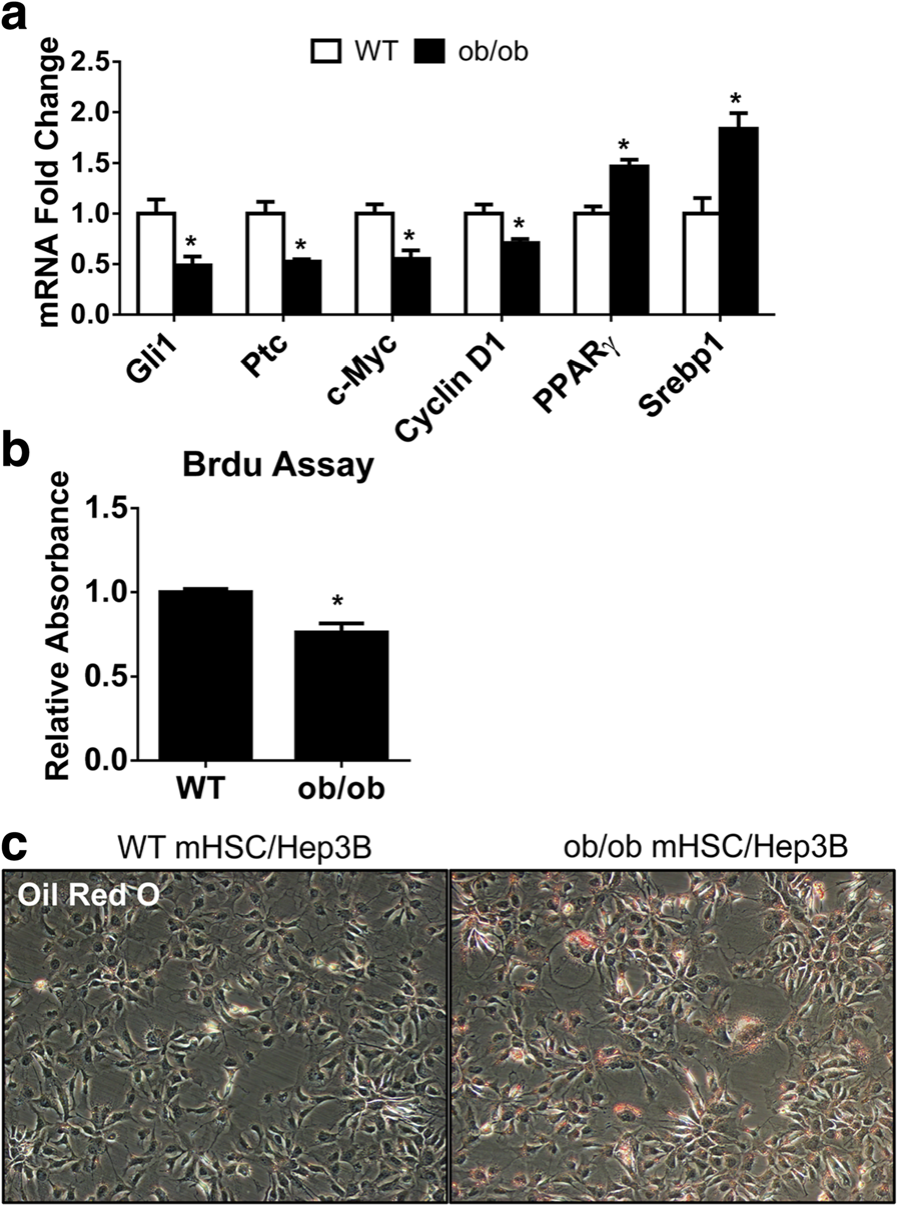
Leptin Deficient HSCs Alter Hepatocyte Phenotype. Primary HSCs isolated from WT or ob/ob mice were cultured alone in Transwell inserts for 4 days. Hep3B cells grown on plastic dishes were then co-cultured with either WT or ob/ob HSC for 3 additional days. **a** qRT-PCR analysis of Hh target genes (Gli1 and Ptc), proliferation genes (c-Myc and Cyclin D1) and lipid associated genes (PPARγ and Srebp1) in Hep3B cells after co-culture with either WT or ob/ob HSC. **b** BrdU assay of proliferation in Hep3B cells co-cultured with either WT or ob/ob HSC. **c** Oil Red O staining was used measure lipid content in Hep3B co-cultured with either WT or ob/ob HSC. (original magnification, ×10). * *p* < 0.05 vs Hep3B co-cultured with WT HSC

HSCs are not the only liver cell type that is dysfunctional in ob/ob mice. For example, ob/ob mice have impaired liver regeneration after partial hepatectomy and ob/ob hepatocytes are fatty and exhibit reduced proliferative activity when exposed to mitogens [6]. We next examined how dysregulated leptin-deficient HSC pericytes, which are inherently different than WT HSCs by having suppressed Hh activity, affected neighboring liver cells. Using a trans-well insert co-culture system, co-culturing Hep3B hepatocyte-derived cells with ob/ob HSCs suppressed Hep3B cell expression of Hh target genes (Gli1 and Ptc), and proliferation genes (c-Myc and cyclin D1), but increased Hep3B expression of lipogenic genes (PPARγ and sterol regulatory element-binding protein 1 (Srebp1)) relative to Hep3B cells that were co-cultured with WT HSCs (Fig. 4a). The functional significance of these gene expression changes was confirmed with BrdU assay and Oil Red O staining (Fig. 4b-c), demonstrating that the Hh-deficient pericytes released signals that caused hepatocytes to become less proliferative and more fatty.

### Leptin supplementation failed to rescue leptin deficient HSCs phenotype

**Fig. 5:**
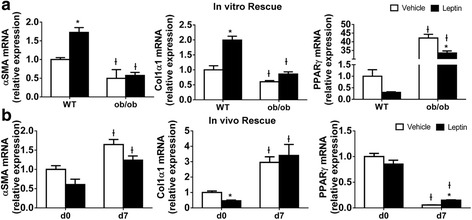
Leptin Treatment Does Not Rescue Leptin-deficient HSCs (**a**) Primary HSCs isolated from either WT or ob/ob mice were cultured in serum-containing medium and treated with leptin (100 ng/ml) or vehicle for 3 days. RNA was isolated, and changes in gene expression of MF markers were evaluated by qRT-PCR. Results were normalized to WT HSCs treated with vehicle. Ɨ *p* < 0.05 vs WT, * *p* < 0.05 vs vehicle. **b** Leptin or vehicle was administrated subcutaneously (100 g/kg body weight per day) to ob/ob mice for 6 days. Primary HSCs were isolated and cultured for 7 days. qRT-PCR analysis of gene expression in freshly isolated and 7-day cultured HSCs. Results were normalized to d0 ob/ob HSCs treated with vehicle. Ɨ *p* < 0.05 vs WT, * *p* < 0.05 vs vehicle

Leptin supplementation has been reported to reverse the “fibrosis-protected” liver phenotype in ob/ob mice [7, 40], so we asked whether leptin supplementation directly effected ob/ob HSCs. Leptin added to day 4 primary HSC cultures further promoted myofibroblastic trans-differentiation of WT HSCs at day 7, evidenced by up-regulation of αSMA and col1α1 but downregulation of PPARγ mRNAs. However, these changes were not observed in ob/ob HSC after leptin treatment (Fig. 5a), despite normal leptin receptor expression in ob/ob and WT HSCs (Fig. 1a). To exclude the possibility that in vitro leptin supplementation might not mimic in vivo supplementation with the hormone, we followed the protocol established previously [7, 40] to administer leptin in vivo. Surprisingly, we failed to observe any improvement in the ob/ob HSC phenotype, either when the cells were examined immediately after isolation or after culture-activation for 7 days (Fig. 5b). Because neither in vitro nor in vivo supplementation with exogenous leptin was sufficient to reverse the fibrosis-protected phenotype of leptin-deficient HSCs, we also examined HSCs from db/db mice to more directly determine the role of reduced leptin signaling in the HSC pathology. db/db mice are hyper-leptinemic but have a mutation in ObRb that prevents leptin from activating Jak2/Stat3. db/db HSC also demonstrated decreased Hh activity and impaired myofibroblastic transdifferentiation compared to WT HSC, (Additional file 1: Figure S2). Collectively, these findings suggested that additional pathways become permanently altered in HSCs in the absence of leptin-ObRb signaling.

### Hh pathway activation improves the phenotype of leptin-deficient HSCs

**Fig. 6:**
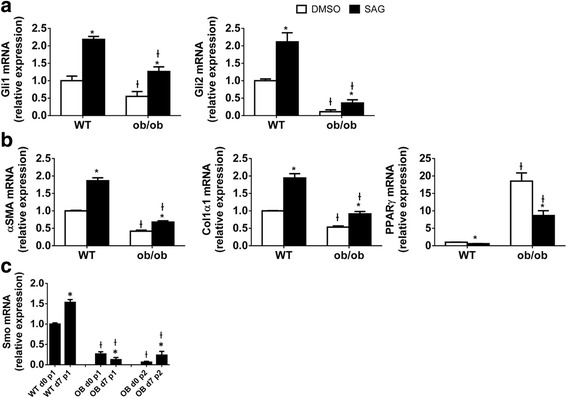
Smoothened Agonist Activates Hh Signaling and Improves Phenotype of Leptin-deficient HSCs. Primary HSCs isolated from either WT or ob/ob mice were cultured in serum-containing medium for 4 days to induce activation and then treated with SAG (0.3 μM, a Smo agonist that activates Hh signaling) or DMSO for 3 additional days. RNA was isolated, and changes in (**a**) Hh target genes (Gli1 and Gli2), (**b**) MF (αSMA and Col1α1) and quiescence (PPARγ) gene expression were evaluated by qRT-PCR. Results were normalized to WT HSCs treated with DMSO. Ɨ *p* < 0.05 vs WT, * *p* < 0.05 vs vehicle. **c** Ultrapure HSCs were obtained by retinoid-based FACS of Nycodenz-gradient purified HSCs from WT and ob/ob mice; RNA was isolated from both freshly FACS-sorted cells (d0) or 7 day cultured FACS-sorted cells (d7), and Smo gene expression was examined by qRT-PCR. Ɨ *p* < 0.05 vs WT, * *p* < 0.05 vs d0

Leptin-deficient HSCs have an abnormally low Hh signaling, so we tried to reverse the fibrosis-protected ob/ob HSC phenotype by increasing Hh pathway activity. First, we treated ob/ob HSCs with Shh ligand, but observed no effect on the expression profile of various myofibroblastic- or quiescence-associated genes (data not shown). In retrospect, this negative result might have been anticipated since expression of Ptc (the Hh receptor) is significantly decreased in ob/ob HSC (Fig. 1b). In contrast, treatment with the drug SAG (which directly activates Smo downstream of Ptc) was able to activate Hh signaling in ob/ob HSCs. SAG increased mRNA expression of two Hh-responsive transcription factors (Gli1 and Gli2), upregulated myofibroblastic genes (αSMA and col1α1), and repressed expression of PPARγ, the quiescence-associated lipogenic gene (Fig. 6a-b). Although expression of these markers improved significantly when ob/ob HSC were treated with SAG, none were restored to levels observed in WT HSC. Therefore, we next examined if inherent differences in Smo expression might be contributing to differential SAG sensitivity of ob/ob and WT HSC. Indeed, qRT PCR analysis demonstrated that Smo mRNA levels were dramatically reduced in the crude (data not shown), ultrapure (P1), and P2 isolates of ob/ob HSC relative to the P1 WT HSC pool (Fig. 6c). These data suggest that Hh activity becomes permanently dysregulated in leptin-deficient HSCs because chronic deficiency of leptin activity suppresses expression of Smo, a critical signaling component of the Hh pathway that must be activated for HSC to become fibrogenic myofibroblasts.

### Genetic deficiency of leptin induces smoothened resistance in adipose-derived mesenchymal stem cells

**Fig. 7:**
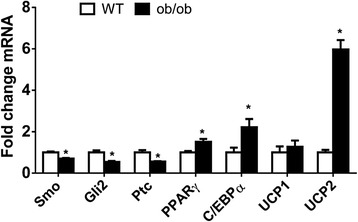
Leptin-deficient Adipose-derived Mesenchymal Stem Cells are Smoothened Resistant. Adipose stromal vascular fractions isolated from WT and ob/ob mice were plated and cultured for 3 weeks to derive homogeneous mesenchymal stem cell populations for RNA isolation. Hh-related genes and markers of adipocyte differentiation were evaluated by qRT-PCR. Results were normalized to WT cells. * *p* < 0.05 vs WT

HSC are members of a larger family of tissue-resident Hh-responsive pericytes that exhibit mesenchymal stem cell characteristics [26, 29, 41, 42]. Adipocyte progenitors derive from Hh-responsive mesenchymal stem cells in the stromal vascular compartment of adult adipose tissue [43]. Having shown that inherited deficiency of leptin causes liver pericytes to acquire a sustained state of Smo resistance that results in an abnormal liver phenotype, we asked if adipose-derived mesenchymal stem cells from ob/ob mice were also Smo-resistant. We reasoned that if this occurred, it would have important implications for the pathogenesis of obesity, as inhibiting Hedgehog signaling is known to promote adiposity [22]. Mesenchymal stem cells were generated from inguinal fat pads of adult ob/ob and WT mice using standard protocols; RNA was prepared and analyzed for differences in the expression of Smo, Hh-regulated genes, and various markers of adipocyte differentiation using qRT-PCR analysis. The results showed that adipose-derived mesenchymal stem cells from adult leptin-deficient mice expressed significantly lower levels of Smo, Gli2, and Ptc, but significantly higher levels of genes involved in white adipose tissue differentiation (e.g., PPARγ, C/EBPα) (Fig. 7). Therefore, unlike normal WAT progenitors that activate the Hh pathway when Jak2-Stat3 signaling is inhibited [18], leptin-deficient adipocyte progenitors with inherently reduced Jak2-Stat3 signaling are unable to do this. Since reducing Hh signaling normally solidifies WAT differentiation [22], we next compared expression of UCP1, a marker of WAT “browning” [44], in control and leptin-deficient adipose-derived progenitors. Adipose progenitors from normal mice were reported to up-regulate UCP1 and exhibit features of “browning” when Jak2-Stat3 signaling was inhibited acutely [18]. However, UCP1 expression was not increased in ob/ob adipose progenitors (Fig. 7), despite the fact that JAK2-Stat3 activity is chronically low when leptin is deficient [45]. The observed inability of Hedgehog-deficient ob/ob progenitors to up-regulate UCP1 is consistent with published evidence that Hh pathway activity was required for adipose progenitors to induce UCP1 after JAK2-Stat3 inhibition [18]. On the other hand, we observed that ob/ob adipose-derived progenitors expressed significantly higher levels of the less potent mitochondrial uncoupler, UCP2, than adipose-derived progenitors from control mice (Fig. 7). These results are consistent with reports that UCP2 expression in WAT is five-fold higher in leptin-deficient ob/ob mice and leptin-resistant db/db mice than in respective littermate controls [46] and correlates with obesity in humans [47].

## Discussion

This study provides evidence that leptin-deficient adult pericytes are inherently different from WT pericytes in both gene expression and function. We found that liver pericytes (a.k.a., hepatic stellate cells, HSC) from leptin-deficient ob/ob mice express normal levels of leptin receptors, despite the fact they are genetically incapable of producing leptin. We also observed that expression of Sonic Hedgehog ligand, receptor, and target genes are all markedly reduced in ob/ob HSCs. Along with inhibited Hh signaling, ob/ob HSCs were found to be less myofibroblastic, proliferative, or migratory, but to express higher levels of quiescence and lipogenic genes and be more prone to senescence. Moreover, we discovered that ob/ob HSCs generate soluble factors that alter the phenotype of neighboring hepatocytes, making the epithelial cells less proliferative but more fatty. Surprisingly, supplementing ob/ob HSCs with leptin failed to rescue normal gene expression profiles despite the fact that these cells express comparable amounts of leptin receptor as WT HSCs. Because we also found similar defects in HSC from db/db mice which are hyperleptinemic but have an ObRb mutation that prevents propagation of leptin-initiated signaling to Jak2/Stat3, these data suggest that other signaling pathways might become permanently altered in HSCs when leptin-ObRb signaling is disrupted chronically.

The phenotypic abnormalities in HSC with mutated leptin- or leptin-receptor genes might have resulted from systemic consequences of life-long leptin insufficiency and/or a cell-autonomous defect in leptin signaling. WT HSCs produce leptin; leptin directly interacts with leptin receptors on HSCs to induce Hh signaling; and the effect of exogenous leptin on MF trans-differentiation of WT HSCs in culture requires a functional Hh pathway [15]. The current study revealed that HSCs in leptin-deficient and leptin-resistant mice develop sustained suppression of various Hh pathway components, including Shh ligand, Ptc (the cell surface receptor for Shh), Smo (the signaling-competent G protein-coupled Hh co-receptor), Gli2 (the Smo-regulated DNA binding protein that controls transcription of Gli1), and Gli1 (a Hh-transcriptional target and pathway effector). Importantly, reduced Smo expression limited the ability of SAG, a direct Smo agonist, to fully restore Hh signaling in ob/ob HSC, but SAG did activate residual Smo sufficiently to induce partial recovery of the myofibroblastic phenotype despite persistent leptin-deficiency. We believe that the progressive “decay” of the Hh signaling pathway in leptin-deficient HSC has broad implications for the pathobiology of leptin deficiency.

Leptin deficiency causes both liver abnormalities and obesity. Like leptin, Shh ligand suppresses white adipose tissue development [22]. In the *Drosophila* fatbody, Hedgehog signaling regulates growth and metabolism, but the Shh that drives this process is produced in another tissue and carried in the circulation in lipid particles to function as a hormone in the fatbody [48]. Shh has been shown to associate with exosomes, microparticles, and lipoproteins in mammals [49], and we previously reported that HSC-derived exosomes and microparticles contain Shh [50]. Those studies also showed that expression of Shh in liver pericytes increases when the cells transdifferentiate into myofibroblasts and that this is accompanied by increases in circulating membrane-associated Shh ligand. Thus, reduced expression of Shh by leptin-deficient pericytes in the liver may reduce exposure of white adipose depots to an important hormone that normally constrains their growth. Further, the current study suggests that the liver might normally be an important source of Shh ligand for adipocytic progenitors because we have preliminary evidence that Shh mRNA expression is several orders of magnitude greater in liver-resident pericytes than in adipose-derived progenitors (data not shown). In any case, the aggregate data suggest the concept that leptin deficiency causes obesity, at least in part, because it reduces adipocyte exposure to Shh and thereby limits activation of the Hh pathway in white adipose depots.

Shh interacts with Ptc on the surface of pericytes and other Hh-responsive cells, and Ptc is necessary for Shh and other Hh ligands to initiate activation of Smo and drive accumulation of Gli transcriptional activators. Thus, leptin-deficient HSC and adipose-derived mesenchymal stem cells exhibit reduced responsiveness to Hh ligands because they are deficient in Ptc. This reinforces the effects of reduced Shh bioavailability in both liver and fat. Indeed, Hh ligand production is induced by liver injury and acts in an autocrine or paracrine fashion to activate many Hh-responsive cell types important in wound healing responses and required for the liver to regenerate [51]. Previously, we reported that Shh is an important effector of HSC trans-differentiation because treating HSCs with antibodies that neutralize Shh blocks that process [52]. Transient hepatic accumulation of HSC-derived myofibroblasts is necessary for the liver to regenerate normally. Earlier work showed that conditional disruption of Hh signaling in HSC-derived myofibroblasts prevented their accumulation after partial hepatectomy and blocked liver regeneration [21]. Liver regeneration is known to be inhibited in ob/ob mice [6] and myofibroblastic trans-differentiation is impaired in leptin-deficient HSC [25]. The present data confirm previous evidence that myofibroblastic HSC generate soluble growth factors for hepatocytes and show, for the first time, that these hepatotrophic actions are defective in leptin-deficient HSC. Indeed, we discovered that leptin-deficient HSC released factors that actually inhibited proliferation of Hep3B cells and caused the liver epithelial cells to become fatty. This insight has potentially important clinical implications because ob/ob livers are fatty and unusually vulnerable to ischemia-reperfusion injury, similar to human steatotic livers [53]. Additional research to characterize the abnormal secretome of leptin-deficient HSC is justified, given the steadily rising prevalence of obesity-related liver disease in the general population. Further study is also needed to determine if leptin deficiency disrupts Hh signaling in other pericyte/stem cell populations, as wound healing responses are highly conserved across tissues [54] and defective skin wound healing is common in human obesity and has already been reported to occur in ob/ob mice [55].

Leptin-deficient HSC are unable to express normal levels of Ptc because they are deficient in Gli1, and Ptc is a Gli1-target gene. Gli1 transcription, in turn, is induced by Gli2 [56]. The cellular level/activity of Gli2 protein is regulated by activated Smo, which inhibits the phosphorylation of Gli2 that targets Gli2 for processing/degradation, and thereby promotes accumulation of Gli2 isoforms that activate gene transcription. We observed that the cellular content of both Gli1 and Gli2 protein increases in WT HSC during trans-differentiation, such that ~90% of myofibroblastic WT HSCs are Gli1-positive and up to 70% WT HSCs harbor both Gli1 and Gli2. In contrast, fewer than 10% of leptin-deficient HSC express Gli1 protein and only about a third are Gli2-positive. The differential accumulation of Gli1 and Gli2 in WT versus ob/ob HSC generally paralleled differences in Smo. We found that WT HSC constitutively express Smo and further induce its expression when they become myofibroblasts. Relative to WT HSC, freshly-isolated ob/ob HSC and db/db HSC express significantly less Smo, and levels of Smo mRNA remain at very low levels even after culture activation. These findings identify leptin-ObRb signaling as a key factor that controls Smo expression. In addition, they suggest that HSC accumulation of Gli1 and Gli2 is mainly dependent upon Smo, with Smo-independent mechanisms playing a relatively minor role. Given that Smo functions as a G protein-coupled receptor to integrate leptin and many other signaling pathways with downstream components/targets of the Hh pathway [57], further research is needed to delineate the mechanisms by which leptin regulates Smo expression.

Our results suggest a novel mechanism that may explain why hepatic regeneration is impaired in ob/ob mice: the profound loss of Smo activity in leptin-deficient HSC. During liver injury, liver-resident pericytes are normally confronted by multiple factors that stimulate them to become myofibroblasts [58]. However, it has been difficult to understand how deficiency of just one of these pro-fibrogenic factors, leptin, conveys a global “myofibroblast/fibrosis-protected” phenotype. On the other hand, earlier work proved that conditionally deleting Smo in adult HSC is sufficient to prevent HSC trans-differentiation, even when cultured on plastic in serum-containing medium [20], and in vivo during various types of acute and chronic liver injury [20, 21]. The new evidence that genetic deficiency of leptin results in loss of Smo in HSC, the key fibrogenic liver cell type, reconciles this paradox.

Further our findings suggest that the inability to transduce myofibroblastic stimuli enhances HSC vulnerability to senescence. This may explain why short-term treatment of adult ob/ob mice with leptin was unable to restore the normal fibrosis-sensitive phenotype in ob/ob HSC in the present study. The fact that leptin-deficient HSC are prone to senescence also has potentially important clinical implications as senescent HSC have been implicated in the pathogenesis of obesity-related hepatocellular carcinoma [59]. While it is unclear whether the incidence of liver cancer is increased in leptin-deficient mice, there is no doubt that the risk for primary liver cancer is increased in humans with obesity-related liver disease, even in the absence of cirrhosis [60]. A previous study suggested that HSC senescence in high fat diet-fed mice is driven by obesity-related changes in the intestinal microbiome [59], and the intestinal microbiome is abnormal in leptin-deficient ob/ob mice and obese humans [61], many of whom are suspected to be leptin-resistant [62]. Further research is needed to determine how the altered intestinal microbiome that occurs during obesity might contribute to inhibition of Smo that occurs in leptin-deficient HSC.

Additional research is necessary to determine whether or not the pericyte abnormalities that result from mutations in the leptin or ObRb genes also occur during diet-induced obesity. This is important because most obese humans are hyper-leptinemic and loss-of-function mutations in ObRb are thought to be relatively uncommon in man. Further, a recent study showed that WT mice with diet-induced obesity retain sensitivity to the central anorexigenic actions of endogenous leptin and gain weight when treated with a leptin receptor antagonist, unlike genetically-obese mice [63]. We could find no reports characterizing pericytes of mice with diet-induced obesity. Like most obese humans, however, most mouse models of chronic diet-induced obesity accumulate relatively few liver myofibroblasts despite prolonged exposure to multiple pro-fibrogenic stressors, raising the intriguing possibility that Hh pathway decay and pericyte senescence may be defects that generally associate with obesity.

## Conclusions

Genetic leptin deficiency suppresses Smo and causes progressive decay of Hedgehog pathway activity in liver-resident pericytes. The resultant inhibition of Hedgehog signaling prevents these cells from being reprogrammed into myofibroblasts, impairing normal regenerative responses to liver injury. In addition, we present novel evidence that the loss of Hedgehog signaling that occurs in the context of genetic leptin deficiency is not restricted to pericytes in liver, but also occurs in multi-potent perivascular cells in white adipose depots. Inhibited Hedgehog activity in multi-potent cells in the stromal vascular compartment of fat depots stimulates these cells to differentiate into white adipocytes and promotes adiposity. Thus, the vulnerable fatty liver obese phenotype that characterizes genetic leptin-deficiency may result from acquired suppression of Smo that limits activation of the Hedgehog pathway in key Hedgehog-responsive cell types. Many other tissues that become dysfunctional during genetic leptin deficiency also harbor Hh-responsive pericytes. The present findings justify further research to determine if these cells also lose Smo activity when deprived of leptin, as this might provide a unifying mechanism to explain the complex phenotype the develops in the context of monogenic leptin-deficient obesity.

## Methods

### Animals

Adult male C57BL6 ob/ob mice, C57BL6 db/db and age/gender-matched wild-type C57BL6 mice were obtained from Jackson Laboratory (Bar Harbor, ME). Animal experiments fulfilled National Institutes of Health and Duke University-Institutional Animal Care and Use Committee (protocol A305-15-12) requirements for humane animal care.

### Cell isolation

Primary HSC were isolated from ob/ob and WT mice (*n* = 56 mice/group) and from db/db mice (*n* = 24) using standard approaches [20]. Cells were pooled from 4 mice/group for each experiment and all experiments were repeated at least twice to assure data reproducibility. Briefly, after *in situ* perfusion of the liver with pronase (Roche) followed by collagenase (Roche), dispersed cell suspensions were layered on a discontinuous density gradient of 8.2 and 15.6% Histodenz (Sigma-Aldrich). The resulting upper layer consisted of >96% HSC. The viability of all cells was verified by phase contrast microscopy and propidium iodide exclusion. The viability of all cells utilized for culture was >98%. Isolated HSC were seeded at a density of 3×10^2^ cells/mm^2^ in DMEM (Invitrogen) supplemented with 10% FBS, streptomycin and penicillin, and non-adherent cells and debris were rinsed away 2 h after initial plating.

Adipose-derived mesenchymal stem cells were isolated following a published protocol [64]. Briefly, inguinal adipose tissue was isolated from 2 mice/group/experiment. Each experiment was repeated twice. Tissues were minced and incubated with collagenase I solution for 1 h at 37 °C with gentle shaking. After digestion, the sample was diluted in DMEM and filtered through 100 and 40 μm filters. Following centrifugation, the pellet was re-suspended in red blood cell lysis buffer, and then centrifuged again. The cells were cultured in DMEM supplemented with 10% FBS, streptomycin and penicillin for 3 weeks to generate a homogeneous mesenchymal cell population.

### Co-culture system

Hep3B cells were obtained from ATCC (Manassas, VA, USA) and cultured in 10% serum-supplemented DMEM medium. To characterize the effects of myofibroblast-hepatocyte interactions in vitro, we used a co-culture system in which primary WT MF-HSCs or ob/ob MF-HSCs and Hep3B cells were cultured for 3 days in a Transwell insert co-culture system, using 0.4-μm pore size polyester (PET) inserts (Corning) as previously described [65].

### Wound-healing assay

Wound-healing assays were performed by scraping cell monolayers as described previously [66]. A reference mark was created on the dish and a time 0 image was acquired. Subsequent images captured matched regions at the indicated times; the size of area lacking cells was quantified (Olympus DP2-BSW software).

### Cell proliferation assay

Cell proliferation was measured using BrdU Cell Proliferation Assay Kit (Cell Signaling, Danvers, MA). Cells were incubated with BrdU for 20 h before the assay.

### Oil Red O staining

To visualize lipid droplets, cells grown on chamber slides were fixed with paraformaldehyde and stained with Oil Red O for 30 min at room temperature, followed by PBS wash.

### Pharmacological manipulation of leptin and Hh signaling

Day 4 primary HSC cultures were treated with Leptin (100 ng/ml; R&D Systems, Minneapolis, MN) or SAG (0.3 μM, Enzo Life Sciences, Plymouth Meeting, PA) as described [15, 67] for 3 days. In separate experiments, 16 ob/ob mice were injected subcutaneously with vehicle (*n* = 8) or Leptin (100 μg/kg body weight per day, *n* = 8) daily for 6 days as described [7]. Primary HSCs were then isolated and cultured for 7 days for qRT-PCR analysis.

### Molecular techniques

qRT-PCR was performed as described [68]. Total RNA was isolated using Qiagen RNeasy Mini Kit and cDNA was synthesized using the SuperScript II First-Strand Synthesis System (Invitrogen). Gene transcripts were quantified by real-time RT-PCR using ABI SYBR Green PCR Master Mix (ThermoFisher) and the StepOnePlus instrument (ABI). Gene expression levels were normalized to reference gene S9, according to the 2^-∆∆Ct^ method. Primers used are listed in Additional file 1: Table S1.

### FACS

All the staining steps were performed in the dark at 4 °C and blocked with BD Fc-Block. For surface staining, cells were incubated for 30 min with antibody in flow cytometry staining buffer (eBioscience) and were washed 3 times with flow cytometry buffer. For intracellular staining, cells were fixed and permeabilized for 20 min with Cytofix/Cytoperm buffer (BD PharMingen) and were washed twice with Perm/Wash buffer (BD PharMingen), followed by incubation with antibody in Perm/Wash buffer for 30 min. LSR II (BD) and FACSDiva software (BD) were used for the acquisition of flow cytometry data, and FlowJo software (TreeStar) was used for analysis. Antibodies used are listed in Additional file 1: Table S2.

### Statistics

All data were expressed as mean ± SEM. Statistical analysis was performed using 2-way ANOVA followed by Tukey's multiple-comparison post hoc test or Student’s *t* test. All analysis was conducted using GraphPad Prism 4 software (GraphPad Software Inc.). Differences with *P* < 0.05 were considered to be statistically significant.

## Additional files

Additional file 1: Figure S1. Sonic Hedgehog Gene Expression in Leptin-deficient HSCs. **Figure S2.** Genetic Leptin Receptor ObRb Deficiency Suppresses Hh Activity in HSCs and Changes HSC Phenotype. **Table S1**. Sequence of primers used in experiments. **Table S2.** Antibodies Used for FACS Analysis (DOC 2609 kb)

## Abbreviations

BrdU: Bromo-deoxyuridine
C/EBPα: CCAAT-enhancer-binding protein type α
Col1α1: Collagen 1 α1 subtype
db/db: Mouse bearing two mutated, inactive leptin receptor ObRb genes
GFAP: Glial fibrillary acidic protein
Gli: Glioma family transcription factor
Hh: Hedgehog
HSC: Hepatic stellate cell
JAK: Janus kinase
MF: Myofibroblast or myofibroblastic-
Ob: Leptin obesity gene
ob/ob: Mouse bearing two mutated, inactive Ob/leptin genes
ObR: Ob/leptin receptor
PDGF: Platelet-derived growth factor
PDGFR: Platelet-derived growth factor receptor
PPARγ: Peroxisome proliferator-activated receptor, type γ
PTC: Patched receptor
Q: Quiescent
qRT-PCR: Quantitative real-time polymerase chain reaction
SAG: Smoothened agonist compound (CAS Number 912545-86-9)
Shh: Sonic hedgehog ligand
Smo: Smoothened receptor protein
SREBP1: Sterol receptor enhancer binding protein 1
UCP: Mitochondrial uncoupling protein
WAT: White adipose tissue
WT: Wildtype mouse
αSMA: α-smooth muscle actin (α-actin-2)
