# Supplementary material for: Loss of pericyte smoothened activity in mice with genetic deficiency of leptin
Source: BMC Cell Biol. 2017 Apr 20;18:20. doi: 10.1186/s12860-017-0135-y (PMC5399438; doi:10.1186/s12860-017-0135-y)
Supplement: Supplementary file 1 — Sonic Hedgehog Gene Expression in Leptin-deficient HSCs. Figure S2. Genetic Leptin Receptor ObRb Deficiency Suppresses Hh Activity in HSCs and Changes HSC Phenotype. Table S1. Sequence of primers used in experiments. Table S2. Antibodies Used for FACS Analysis (DOC 2609 kb) [file 12860_2017_135_MOESM1_ESM.doc]

**Supplementary Figure 1. Sonic Hedgehog Gene Expression in Leptin-deficient HSCs.** Primary HSCs were isolated from WT mice or ob/ob mice, and cultured on plastic dishes in serum-containing medium for 2 hours (d0) or 7 days (d7). Isolated RNA was subjected to qRT-PCR using primers for the Sonic hedgehog gene product. Ɨ p<0.05 vs WT, * p<0.05 vs vehicle.


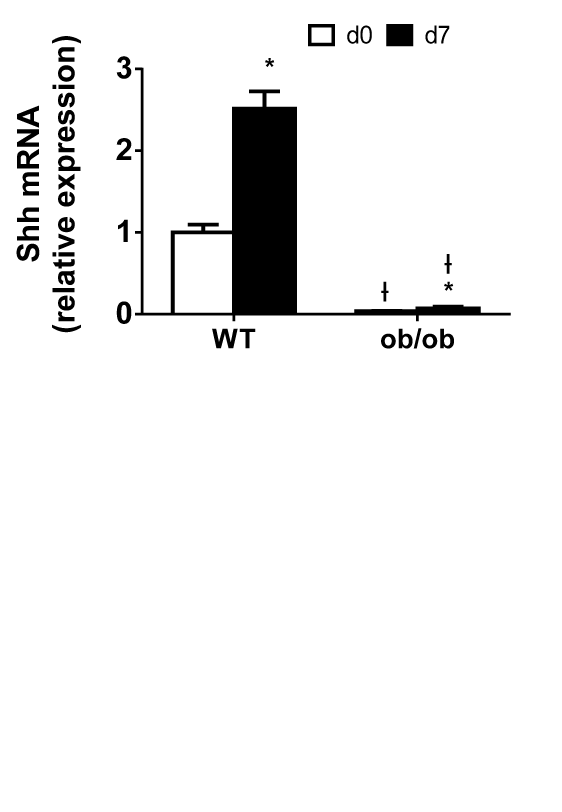


**Supplementary Figure 2. Genetic Leptin Receptor ObRb Deficiency Suppresses Hh Activity in HSCs and Changes HSC Phenotype.** Primary HSCs were isolated from WT mice or db/db mice, and cultured on plastic dishes in serum-containing medium for 2 hours (d0) or 7 days (d7). (A) FACS analysis of Hh target gene product (Gli2), (B) qRT-PCR and (C) FACS analysis of MF genes (αSMA, Col1α1, and PDGF), and (D) qRT-PCR analysis of quiescence genes (PPARγ, CD36, and GFAP) in quiescent d0 and myofibroblastic d7 HSCs. Results were normalized to WT d0 cells. p<0.05 vs WT, * p<0.05 vs d0. n=3. Red lines indicate Isotype controls. (E) Lipid content of cultured HSC at indicated days was examined by Oil Red O staining. (original magnification, ×10) (F) Senescence was examined by β-galactosidase staining (blue) of HSCs cultured for 1, 4 and 7 days. (original magnification, ×10)


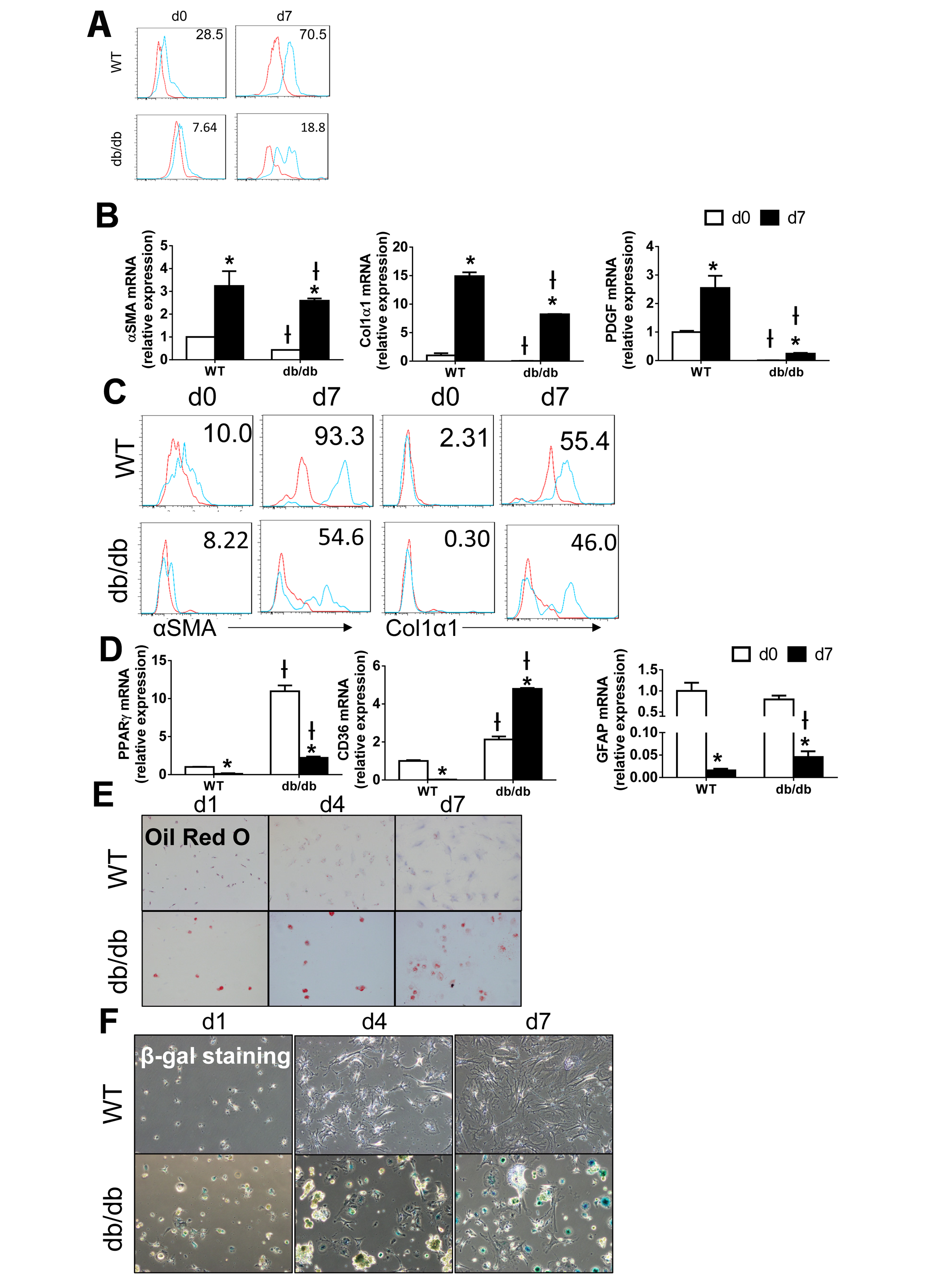


**Supplementary Table 1: Sequence of primers used in experiments**

Mouse Primers

| **Product** | **Forward sequence** | **Reverse Sequence** |
| --- | --- | --- |
| S9 | GACTCCGGAACAAACGTGAGGT | CTTCATCTTGCCCTCGTCCA |
| Col1α1 | GAGCGGAGAGTACTGGATCG | GCTTCTTTTCCTTGGGGTTC |
| αSMA | GATGAAGCCCAGAGCAAGAG | CTTTTCCATGTCGTCCCAGT |
| GFAP | agaacaacctggctgcgtat | ccagcgattcaacctttcTC |
| PPARγ | AGGCCGAGAAGGAGAAGCTGTTG | TGGCCACCTCTTTGCTCTGCTC |
| LRAT | GGCACAGGGAAGAACC | TTAGATGGGCGACACG |
| e-cadherin | ACCTCTGGGCTGGACCGA | CCTGATACGTGCTTGGGTTGAA |
| CD146 | TGTGCGTCTTCTTGTTCGCT | ACTTGGCTGAAGTTGCCTGA |
| PDGFRβ | GACTACCTGCACCGGAACAA | GTCCAACATGGGCACGTAA |
| Gli1 | CCTCCTCCTCTCATTCCACA | CTCCCACAACAATTCCTGCT |
| Gli2 | CCCCATCACCATTCATAAGC | CTGCTCCTGTGTCAGTCCAA |
| Shh | GAAGATCACAAGAAACTCCGAACGA | CCTCTGAATGATGGCCGTCCT |
| Smo | GTCACTGTCCTCATCCCCTT | CAAAACAGATGCCACTCACG |
| Ptc1 | ATGCTCCTTTCCTCCTGAAACC | TGAACTGGGCAGCTATGAAGTC |

Human Primers

| **Product** | **Forward sequence** | **Reverse Sequence** |
| --- | --- | --- |
| β-actin | TGGCATCCACGAAACTACCT | ACGGAGTACTTGCGCTCAG |
| PPARγ | GAAGTTCAATGCACTGGAATTAGAT | ATCTGTCTGAGGTCTGTCATTTTCT |
| c-Myc | GCCAAGAGGGTCAAGTTGGA | CAAGACGTTGTGTGTTCGCC |
| CyclinD1 | ATGCCAACCTCCTCAACGAC | GGACCTCCTTCTGCACACAT |
| Srebp1 | CTGACCGACATCGAAGGTGA | CCAGCATAGGGTGGGTCAAA |
| Gli1 | GACGCCATGTTCAACTCGAT | GCCGGACATGAGGTTAGCTT |
| Ptc | ACTCCCAAGCAAATGTACGAGCACT | AGACAGGCATAGGCGAGCATGA |

| **Supplement Table 2: Antibodies Used for FACS Analysis** | | | |
| --- | --- | --- | --- |
| **Name** | **Host / isotype** | **Source** | **Catalog No.** |
| αSMA | Rabbit IgG | Abcam | ab32575 |
| Col1α1 | Rabbit IgG | Abcam | ab292 |
| Gli1 | Rat IgG2a | R&D Systems | AF3324 |
| Gli2 | Rabbit IgG | Genway Biotech | 18-732-292462 |
| Leptin | Rabbit IgG | Abcam | ab16227 |
| Leptin R | Goat IgG | R&D Systems | AF797 |
| PDGF | Rabbit IgG | Abcam | ab23914 |
| Ptc | Goat IgG | R&D Systems | AF4105 |
